# Plasmonic ELISA for Sensitive Detection of Disease Biomarkers with a Smart Phone-Based Reader

**DOI:** 10.1186/s11671-018-2806-9

**Published:** 2018-12-05

**Authors:** Quanli Yang, Ruitian Cai, Wei Xiao, Zengfeng Wu, Xia Liu, Yan Xu, Miaomiao Xu, Hui Zhong, Guodong Sun, Qihui Liu, Qiangqiang Fu, Junjian Xiang

**Affiliations:** 1 Institute of Biotranslational Medicine, Jinan University, Guangzhou, 510632 People’s Republic of China; 2 Department of Bioengineering, Guangdong Province Key Laboratory of Molecular Immunology and Antibody Engineering, Jinan University, Guangzhou, 510632 People’s Republic of China; 3 Department of Orthopedics, First Affliated Hospital, Jinan University, Guangzhou, 510632 People’s Republic of China

**Keywords:** Serum myoglobin, Acute myocardial infarction, Plasmonic immunoassay, Smart phone

## Abstract

Serum myoglobin is one of the earliest markers for the diagnosis of acute myocardial infarction. It is, therefore, critical to develop a point-of-care testing technology for myoglobin detection. In this work, we reported a sensitive plasmonic immunoassay-based on enzyme-mediated localized surface plasmon resonance change of gold nanorods for the point-of-care testing detection of myoglobin. In addition, we developed a novel plasmonic immunoassay reader using the ambient light sensor of smart phone to increase the accessibility and utility of the plasmonic immunoassay. The linear detection range of gold nanorods-based plasmonic immunoassay for myoglobin detection was 0.1–1000 ng mL^−1^ and the limit of detection was 0.057 ng mL^−1^. Myoglobin in serum samples was also analyzed by the plasmonic immunoassay. The results were significantly correlated with those of conventional enzyme-linked immunosorbent assay. The plasmonic immunoassay, coupled with smart phone-based reader, could be widely used for point-of-care testing application of acute myocardial infarction, especially in the regions with limited technological resources.

**Electronic supplementary material:**

The online version of this article (10.1186/s11671-018-2806-9) contains supplementary material, which is available to authorized users.

## Introduction

Acute myocardial infarction (AMI) is the medical name for a heart attack that occurs when the blood flowing to the heart muscle is abruptly cut off, leading to tissue damage [1]. Symptoms of AMI include severe and persistent post-sternal pain, arrhythmia, shock, and heart failure, which could be fatal [2]. AMI is one of the most common diseases in Europe and America. Approximately, 1,500,000 people suffer from myocardial infarction every year in the USA alone. An obvious increase trend of AMI has also been observed in China in recent years, with at least 500,000 new patients per year. Point of care test of biomarker is of great significance for the early monitoring and treatment of AMI. Serum myoglobin (Myo) increases in 1–2 h after an AMI and reaches peak value at 6–9 h. Myo is believed to be one of the earliest serum markers for early diagnosis of AMI [3–5].

Recently, plasmonic immunoassays have been developed by combining traditional enzyme-linked immunosorbent assay (ELISA) and nanomaterials [6, 7]. Plasmonic immunoassays have been widely used in clinical diagnosis [8], environmental pollution monitoring [9], and food safety detection [10]. Compared to other immunoassays, plasmonic immunoassays are highly sensitive and allow for naked-eye readout without the use of sophisticated instruments. Metal nanoparticles, such as gold and silver nano-materials, are commonly used in plasmonic nanosensors, due to their excellent localized surface plasmon resonance (LSPR). In plasmonic immunoassays, the enzyme-labeled antibody catalyzes its substrates to generate a product that triggers the aggregation or shape change of the nanomaterials. Chen’s group [11] reported a plasmonic immunoassay for pathogen detection using acetylcholinesterase (AChE)-catalyzed hydrolysis reaction to induce the aggregation of gold nanoparticles (AuNPs). The sensitivity of the plasmonic immunoassay is comparable to RT-PCR. However, the robust, ultra-rapid, and highly stable AuNPs aggregation in colorimetric assay remains a challenge due to the aggregation procedure of AuNPs being dynamic [12, 13]. Another kind of plasmonic immunoassay is based on inducing shape change of plasmonic nanoparticles. Triangular silver nanoprisms (AgNPRs) etching-based plasmonic biosensors were reported for the detection of cancer biomarkers, which uses hydrogen peroxide (H_2_O_2_) produced by glucose oxidase (GOx) for AgNPR etching [14, 15]. However, in order to quantitatively test a molecular target, relatively sophisticated and bulky instruments for measuring spectrum of nanomaterials are necessary along with the plasmonic immunoassays. The inconvenience associated with these instruments makes them inapplicable for point of care analysis. Therefore, there is an urgent need to develop a portable, inexpensive, and easy-to-use plasmonic immunoassay reader for a point-of-care testing (POCT) diagnosis.

Gold nanorods (AuNRs) have been widely adopted in biosensors [16–18], plasmonic imaging [19], tumor photothermal therapy [20], and photodynamic therapy [21] due to their unique physical, optical and electronical properties [22–24]. In this work, we reported a sensitive plasmonic immunoassay based on the enzyme-mediated LSPR change of AuNRs for the detection of Myo. In order to facilitate POCT diagnosis of AMI, we developed a novel plasmonic immunoassay reader using the ambient light sensor (ALS) of a smart phone. The high correlation between the results obtained from the plasmonic immunoassay and those from traditional ELISA on serum specimens demonstrated the application potentials of this new method for early POCT diagnosis of AMI, especially in the regions with limited technological resources.

## Materials and Methods

### Materials and Reagents

Myo (from human heart tissue) was purchased from Abcam (Cambridge, UK). Anti-myo monoclonal antibodies (mAb1 and mAb2) were produced in our lab (Additional file 1). Silver nitrate (AgNO_3_, 99.8%) and sodium borohydride (NaBH_4_) were purchased from Sinoreagent (Shanghai, China). The hydrogen peroxide (H_2_O_2_, 30 wt%) and H_2_SO_4_ were purchased from GZ chemical reagent factory (Guangzhou, China). Light-emitting diode (LED, 850 nm) was purchased from Shenzhen OCtai Co., Ltd. (Shenzhen, China). Chloroauric acid (HAuCl_4_), TMB (3,3′,5,5′-tet-ramethylbenzidine), Tween-20, and cetyltrimethylamm-onium bromide (CTAB) were purchased from Amresco (Houston, TX, USA). Glucose oxidase type VII from *Aspergillus niger* (GOx), horseradish peroxidase type VI (HRP), and bovine serum albumin (BSA) were purchased from Sigma-Aldrich (Saint Louis, MO, USA). Deionized water (Milli-Q grade, Millipore) with a resistivity of 18.2 MΩ cm was used throughout this study. Serum samples were collected from the Guangzhou Overseas Chinese Hospital (Guangzhou, China).

### Apparatus

The LSPR spectra of AuNRs in 96-well plates were collected by a Synergy H1 Hybrid Multi-Mode Microplate Reader (Bio-Tek Instruments, Inc. USA). The absorbance of the HRP-based ELISA was measured at 450 nm using a MK3 microplate reader (Bio-Tek Instruments, Inc. USA). Characterization of AuNRs was performed with a PHILIPS TECNAI-10 transmission electron microscope (TEM) operating at an acceleration voltage of 120 kV. The samples for TEM measurements were prepared by depositing one drop of aqueous dispersion onto a copper grid coated with thin films of carbon, and the solvent was removed by evaporation in air. A 3D printer was purchased from the SHINING 3D (Hangzhou, China). A HUAWEI P9 smart phone (Shenzhen, China) was chosen as the basic smart phone for the plasmonic immunoassay reader.

### Design of the Smart Phone-Based Plasmonic Immunoassay Reader

The design of the smart phone-based plasmonic immunoassay reader was created with software (Solidworks 2014), and then processed by the software, 3D star. For printing setup, print mode was set to quality and the supporting way was configured to internal and external support. A free smart phone application, Light Meter, was utilized to display measured results on the screen. In this work, the plasmonic immunoassay reader is running on an android (open source) phone. This reader also can be used on iPhone, if the user applied an iOS version software (Light Meter).

### Synthesis of AuNRs

AuNRs were prepared by seed gold-mediated growth [25]. Gold seed preparation: fresh 0.01 M sodium borohydride in 0.01 M sodium hydroxide was prepared. Then, 600 μL of sodium borohydride solution were added to a HAuCl_4_ solution (0.25 mM) in 10 mL 0.1 M CTAB under stirring (300 rpm min^−1^). The color of the gold seed changed from greenish to light brown. Synthesis of nanorods: AgNO_3_ (70 μL, 0.1 M) solution was added to 10 mL HAuCl_4_ solution (0.5 mM) in 0.1 M CTAB. Subsequently, 140 μL of ascorbic acid (0.0788 M) were added under stirring (300 rpm min^−1^). Finally, 12 μL of gold seed were added, and the solution was mixed under stirring (300 rpm min^−1^) for 12 h before use.

### Procedure of AuNRs-Based Plasmonic Immunoassay for Myo Detection

For the plasmonic immunoassay, to prepare the 96-well polystyrene plates with anti-Myo antibody 1 (Ab1), diluted Ab1 was incubated in 96-well polystyrene plates at 4 °C overnight. After three washes with PBST, the 96-well polystyrene plates were blocked with blocking buffer (1 mg mL^−1^ BSA in PBST) at 37 °C for 1 h. Then the 96-well polystyrene plates were washed three times with wash buffer, and stored at − 20 °C. The Anti-Myo antibody 2 labeled with GOx (GOx-Ab2) was prepared by following the procedures showed in the Additional file 1.

For Myo detection, different concentrations of Myo solutions (100 μL) were added to the Ab1-coated 96-well polystyrene plates. After incubation for 1 h, the plates were washed three times with PBST buffer, and then 0.01 mg mL^−1^ GOx-Ab2 was added and incubated at 37 °C for another 1  h. Then, the plates were washed three times with PBST buffer, and 50 μL glucose (0.5 mM) was added and incubated at 37 °C for 30 min. Subsequently, the supernatant was mixed with 50 μL citrate buffer (40 mM, pH 4.0) containing AuNRs ([Au0] 0.24 mM), CTAB (12.5 mM), and HRP (3 μM), and incubated for 30 min. The corresponding LSPR spectrum of the AuNRs was collected by a commercial microplate reader and the transmitted light intensity (850 nm) of the AuNRs was measured by the smart phone-based plasmonic immunoassay reader. The calibration curve of the plasmonic immunoassays for Myo was constructed by fitting measured transmitted light intensity to the related Myo concentration. For detection of Myo, serum samples were diluted ten times using PBS buffer. Then, 100 μL of the diluted serum samples were added to the Ab1-coated 96-well polystyrene plates. The concentration of Myo was tested as described above. Each value presents the mean from three replicates.

The procedure for HRP-based ELISA is shown in the Additional file 1.

### Data Analysis Method

Linear regression analysis was processed with origin 9.0. All experiments were repeated three times independently. Each value presents the mean from three replicates.

## Results and Discussion

### Principle of the AuNRs-Based Immunoassay for Myo Detection

**Fig. 1:**
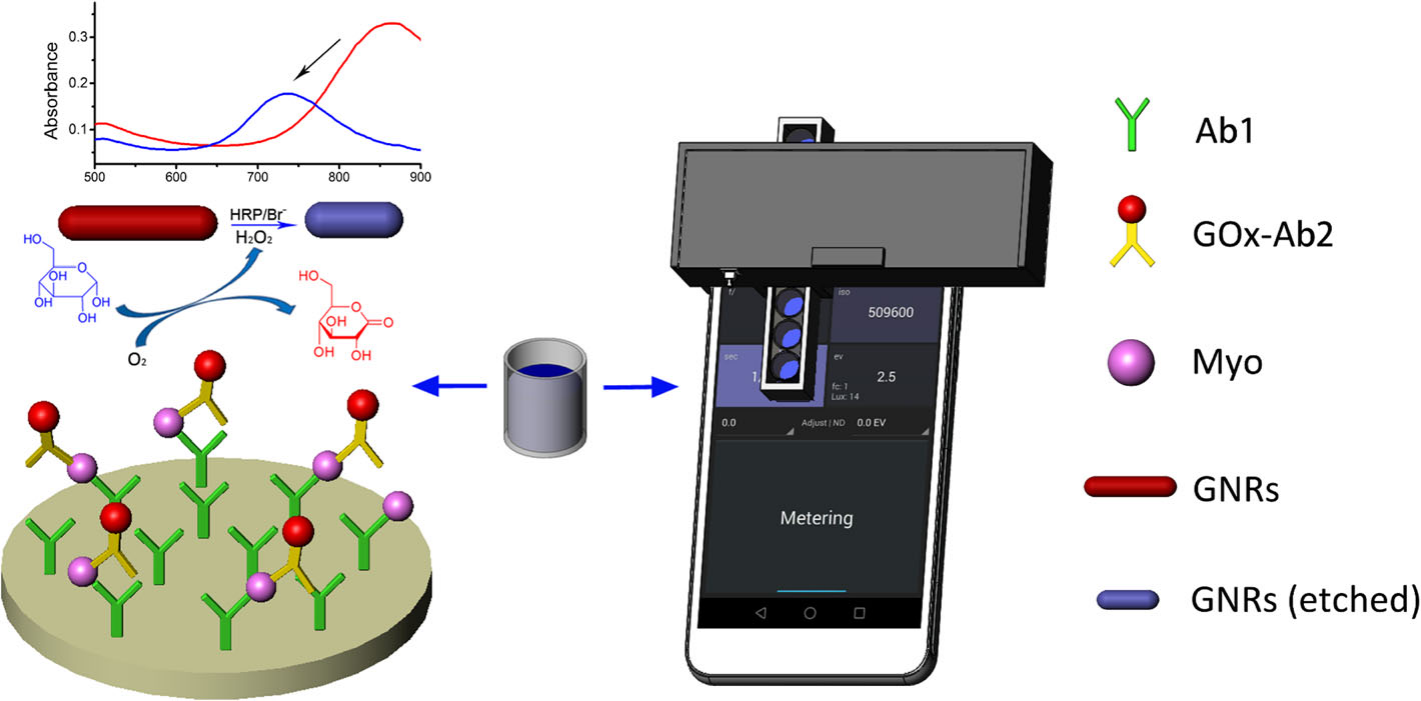
Schematic diagram of the AuNRs-based plasmonic immunoassay for Myo detection

The plasmonic immunoassay combines sandwich immunoassay with the plasmonic characteristic of AuNRs. Ab1 and Ab2 were conjugated with glucose oxidase (GOx-Ab2) (Fig. 1). Upon binding, the GOx-labeled Ab2 could catalyze its substrate glucose to generate gluconic acid and hydrogen peroxide (H_2_O_2_). The H_2_O_2_ acts as an oxidant to etch the AuNRs under certain concentration of HRP and Br^−^, which leads to a substantial blue shift in the SPR spectrum of AuNRs and a decrease in the absorbance of AuNRs at 850 nm. In the plasmonic immunoassay, the amount of GOx is proportional to the target concentration. The degree of blue shift in the SPR spectrum of AuNRs and absorbance reduction of AuNRs at 850 nm was positively correlated to target concentrations. The results of plasmonic immunoassay could be qualified by using a microplate reader to measure the blue shift of AuNRs SPR spectrum or by using the smart phone reader to measure the change in the absorbance of AuNRs at 850 nm.

### Optimization of Plasmonic Immunoassay for Myo Detection

**Fig. 2:**
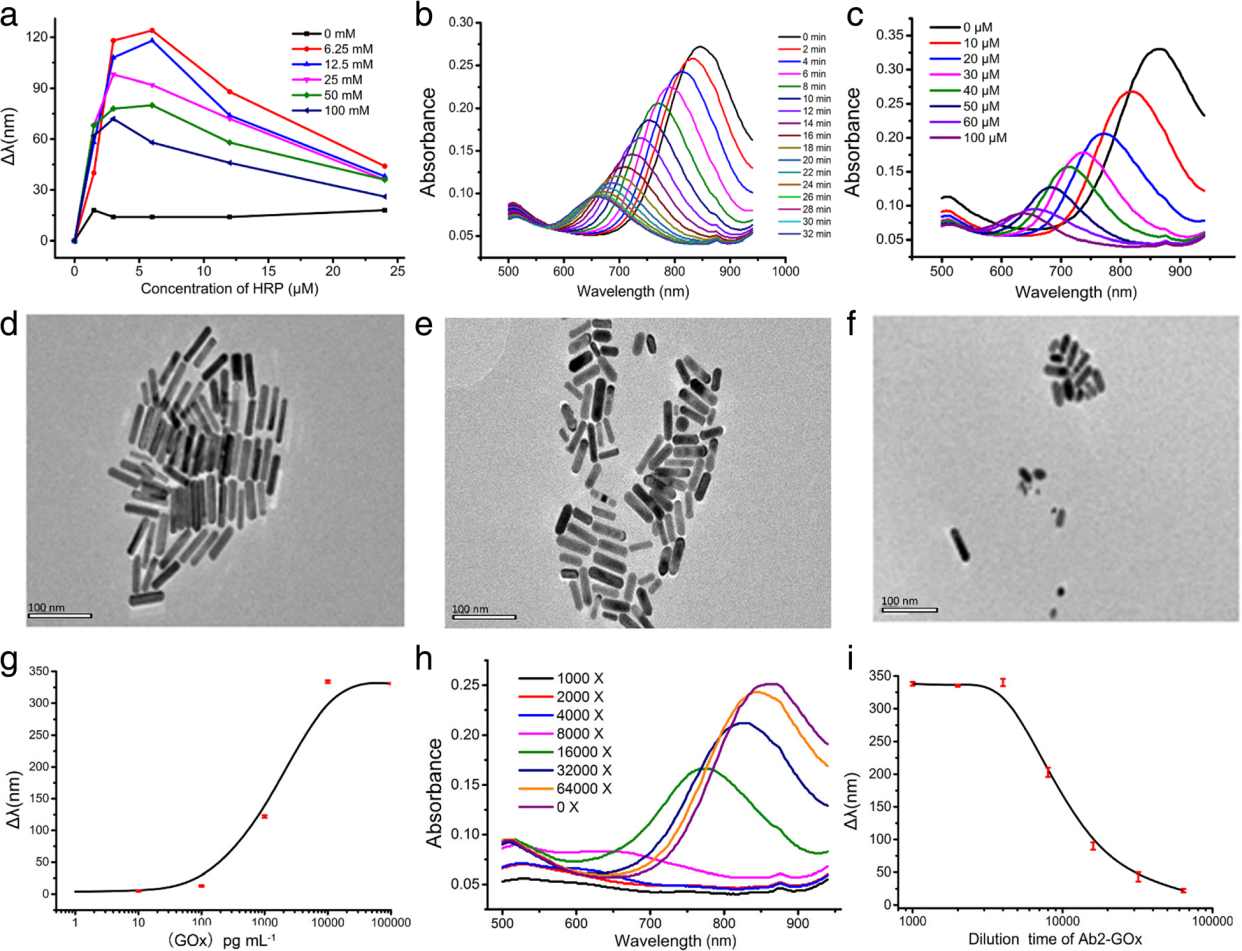
Optimization and characterization of AuNRs based on plasmonic immunoassay for Myo detection. **a** Optimization of HRP and CTAB concentration in AuNRs based on plasmonic immunoassay. Different color lines represent different concentrations of CTAB, among which 6.25 mM CTAB and 1.5 μM HRP were preferred. **b** Optimization of time for H_2_O_2_ etching AuNRs, for which 1.5 μM HRP, 6.25 mM CTAB, and 100 μM H_2_O_2_ contained in citrate buffer (20 mM, pH 4.0) for 30 min was preferred. **c** LSPR spectrum of AuNRs with the addition of 50 μL of varying concentrations of H_2_O_2_. **d**–**f** TEM images of AuNRs etched by different concentrations of H_2_O_2_ (0 μM, 10 μM, and 100 μM) for 30 min. **g** LSPR shift of AuNRs under different concentrations of GOx. **h** LSPR spectrum of AuNRs in the presence of GOx-Ab2 at different dilution ratios. **i** LSPR spectrum shift of AuNRs in the direct plasmonic immunoassay coated with different concentrations of Myo. Each value presents the mean from three replicates

HRP and CTAB concentrations directly affect the detected results. In this work, AuNRs were etched by a solution that contained 100 μM of H_2_O_2_, and different concentrations of HRP and CTAB in citrate buffer (40 mM, pH 4.0), from which LSPR shift of AuNRs was recorded. In this study, 1.5 μM HRP and 6.25 μM CTAB were selected due to the maximal LSPR shift of AuNRs observed at these concentrations (Fig. 2a). At the optimized HRP and CTAB concentrations, AuNPs were etched by 100 μM H_2_O_2_ in citrate buffer (20 mM, pH 4.0). LSPR spectrum of AuNRs was blue shifted and the absorbance of AuNRs at 850 nm was decreased with time (Fig. 2b). After 30 min, LSPR of AuNRs stable. Therefore, 30 min was selected as the time for H_2_O_2_ etching of AuNRs. AuNPs were etched by different concentrations of H_2_O_2_. AuNRs LSPR spectrum was blue shifted with decreasing H_2_O_2_ concentration (Fig. 2c). TEM images of AuNRs showed that with increasing H_2_O_2_ concentration, the shape of AuNRs changed from rectangle to ellipse (Fig. 2d–f). These results demonstrated that AuNRs LSPR spectrum was dependent on the concentration of H_2_O_2._

GOx could catalyze glucose to produce H_2_O_2_, which could etch AuNRs. In this work, 0.5 mM glucose was catalyzed by different concentrations of GOx, and the produced H_2_O_2_ was used to etch AuNRs (Fig. 2g). When the concentration of GOx was at 100 pg mL^−1^ (6.66 × 10^−11^ mol L^−1^), the LSPR spectrum of AuNRs showed an obvious blue shift, suggesting the high sensitivity of the AuNRs-based plasmonic immunoassay. GOx was conjugated with Ab2 and then diluted to different concentrations. The catalytic activity of GOx-Ab2 was validated by decomposed GOx and etched AuNRs. The LSPR spectrum of AuNRs was blue shifted with increasing GOx-Ab2 concentration (Fig. 2h), which indicated that GOx-Ab2 maintains good catalytic activity. In addition, Myo was coated on microplates and incubated with GOx-Ab2. After 30 min, GOx-Ab2 was washed three times with PBST buffer. Glucose was then added to the microwell, followed by the addition of AuNRs to the glucose solution after 30 min. The blue shift of the LSPR spectrum of AuNRs increased with increasing GOx-Ab2 concentration (Fig. 2i), demonstrating that Ab2 maintains its immunologic activity, while GOx maintains its catalytic activity.

### Smart Phone-Based Plasmonic Immunoassay Reader for On-site Myo Detection

**Fig. 3:**
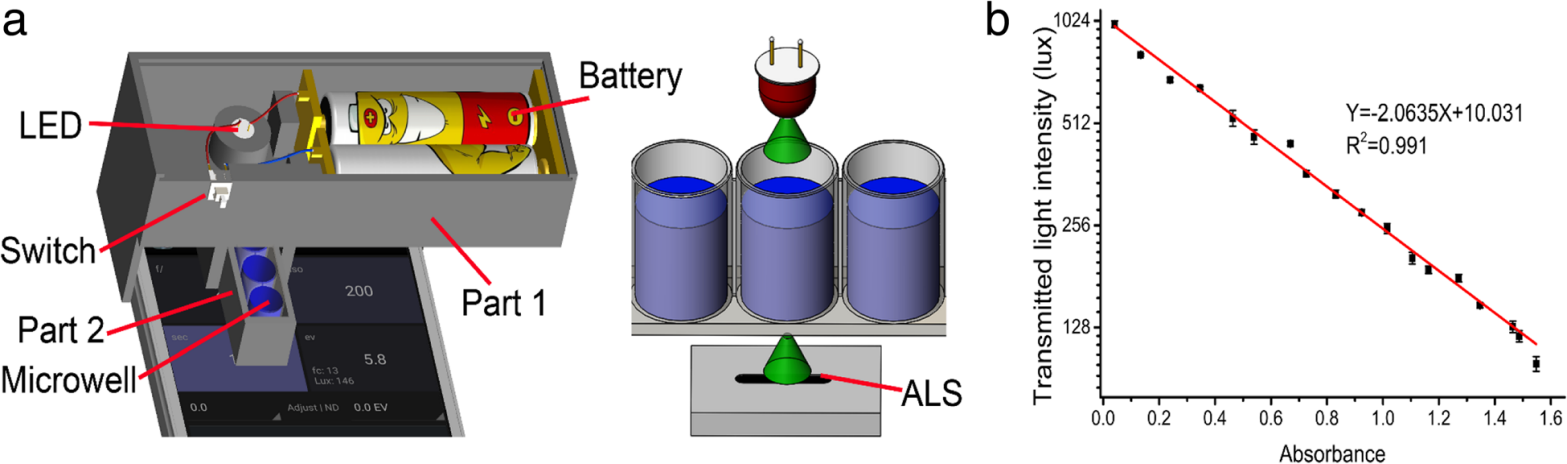
Mechanism of smart phone-based plasmonic immunoassay reader. **a** Schematic of the 3D-printed accessory of smart phone-based plasmonic immunoassay reader. Transmitted light intensity of AuNRs was measured by ALS of the smart phone and the value was displayed onto a screen. **b** The correlation between results from the smart phone based plasmonic immunoassay reader and those from the commercial microplate reader. Each value presents the mean from three replicates

Commonly reported plasmonic immunoassays are interpreted quantitatively by a commercial microplate reader or a spectrometer, which limits their utility in resource-limited regions. In order to improve the accessibility of our plasmonic immunoassay, we prepared a smart phone-based plasmonic immunoassay reader that relies on ambient light sensor (ALS) of a smart phone to measure transmitted light intensity of AuNRs. In most smart phones, ALS is a default configuration used to automatically adjust the light intensity of the screen depending on various circumstances. We previously reported using ALS in colorimetric assay reader [26–28]. The principle and instruction for the plasmonic immunoassay reader were documented in the previous publication [29]. The 3D-printed plasmonic immunoassay reader consists of two parts: part 1 (100 mm × 40 mm × 40 mm) could be fixed on a smart phone to supply a stable light source powered by two batteries (1.5 V), and part 2 (76 mm × 13 mm × 12 mm) could be used to host microwells. Once the plasmonic immunoassay was completed, the microwell was assembled onto part 2, as shown Fig. 3a, and then part 2 was fixed onto part 1. Part 1 was then attached to the ALS of the smart phone. In this design, the LED was aligned with the ALS of the smart phone. Once the switch was turned on, the light from the LED was transmitted across AuNRs and measured by the ALS. The transmitted light intensity of each microwell could be read by sliding part 2. Through the Android application Light Meter, the measured results could be presented on the screen of a smart phone. In the plasmonic immunoassay, the maximum absorption spectrum of AuNRs was 850 nm, and with increasing H_2_O_2_ concentration, the absorbance of AuNRs at 850 nm was gradually decreased. Therefore, 850 nm was selected as the wavelength of the exciting light of the LED in the plasmonic immunoassay reader. The total cost of the smart phone-based plasmonic immunoassay reader was about two dollars. To compare measured results from the smart phone-based plasmonic immunoassay reader and those from commercial microplate reader, the transmitted light intensity and absorbance of AuNRs in microwell were measured. Results obtained from these devices were fitted and showed a 99.1% correlation (Fig. 3b), indicating that the smart phone-based plasmonic immunoassay reader was a comparable tool in terms of accuracy.

### Analytical Performance of the Plasmonic Immunoassay for Myo Detection

**Fig. 4:**
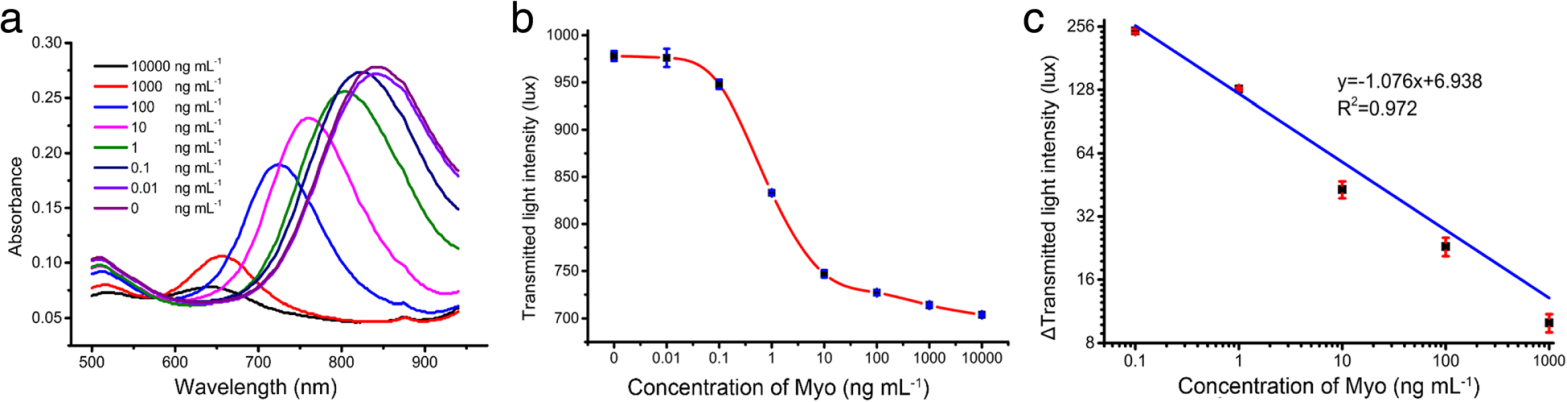
Plasmonic immunoassay for Myo detection. **a** LSPR peak shifts of AuNRs at different concentrations of Myo. **b** Absorbance of AuNRs for the detection of different concentrations of Myo. **c** Calibration line of plasmonic immunoassay for Myo detection as read by smart phone-based reader. Each value presents the mean from three replicates

For the performance of AuNRs-based plasmonic immunoassay, different concentrations of Myo were analyzed. LSPR spectrum of AuNRs was recorded by a commercial spectrometer, and the transmitted light intensity of LSPR spectrum of AuNRs was measured by the smart phone-based plasmonic immunoassay reader. With increasing Myo concentrations, LSPR spectrum of AuNRs was blue shifted and the absorbance of LSPR spectrum of AuNRs was decreased (Fig. 4a). LSPR blue shift of AuNRs was used for quantitative analysis of Myo concentration. When the Myo concentration was at 0 pg mL^−1^, the LSPR blue shift of AuNRs was 0 nm. With increasing Myo concentrations, the LSPR peaks of AuNRs were blue shifted (Additional file 1: Figure S1). The measured transmitted light intensities were employed to quantify the concentrations of Myo. The linear detection range of AuNRs based on plasmonic immunoassay quatified by LSPR spectrum blue shift was 0.1–1000 ng mL^−1^ (Additional file 1: Figure S2-S3) with the limit of detection at 57.81 pg mL^−1^. The measured transmitted light intensity of AuNRs decreased with increasing Myo concentrations (Fig. 4b). The measured transmitted light intensities were employed to quantify the concentrations of Myo. The linear detection range of plasmonic immunoassay quantified by transmitted light intensity of AuNRs was 0.1–1000 ng mL^−1^ (Fig. 4c) with the limit of detection at 64.13 pg mL^−1^. AMI was defined as serum concentration of Myo higher than 90 ng mL^−1^. For detection of Myo in clinical samples, serum was diluted ten times prior to analysis to improve the consistency of the measured results.

### Comparison of the Plasmonic Immunoassay and ELISA

**Fig. 5:**
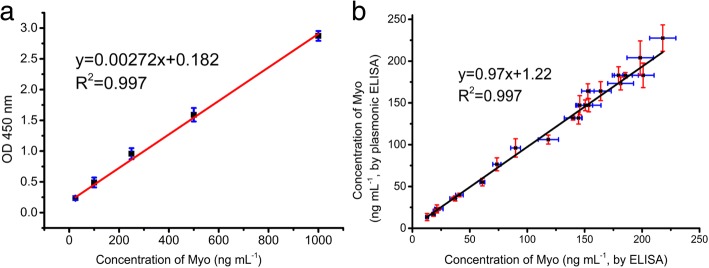
Comparison of the plasmonic immunoassay and conventional ELISA for Myo detection. **a** Calibration line of ELISA for Myo detection. **b** Comparison of the plasmonic immunoassay and conventional ELISA in testing serum samples. The transverse axis represents results from ELISA and the vertical axis represents results from the plasmonic immunoassay. Each value presents the mean from three replicates

ELISA is one of the most widely used techniques in clinical diagnosis. In this work, we compared the detection performance of ELISA and the plasmonic immunoassay for Myo analysis. The same antibodies and antigens were used in both methods. The linear detection range of ELISA was 25–1000 ng mL^−1^ and the LOD was 22.7 ng mL^−1^ (Fig. 5a, Additional file 1: Figure S4). Compared with ELISA, the plasmonic immunoassay was more sensitive and exhibited a wider detection range. In addition, to demonstrate the feasibility of using the plasmonic immunoassay for clinical application, Myo in clinical serum samples was measured by the plasmonic immunoassay and ELISA. Results of plasmonic immunoassay were read by a commercial microplate reader and smart phone-based plasmonic immunoassay reader, respectively. The results from these two methods were well-correlated (Fig. 5b), indicating that the AuNRs-based plasmonic immunoassay could be used for clinical AMI diagnosis.

## Conclusions

By taking advantage of the unique optical properties of AuNRs, we successfully developed a plasmonic immunoassay for detecting acute myocardial infarction in clinical samples. To improve the utility of the immunoassay in on-site testing, we prepared a smart phone-based plasmonic immunoassay reader that relies on ALS of the smart phone to measure transmitted light intensity of AuNRs. The limit of detection of the AuNRs-based plasmonic immunoassay read by the smart phone reader was 0.057 ng mL^−1^. This biosensor was more sensitive than conventional ELISA, making it a promising platform for biomedical applications. In addition, by using the smart phone-based plasmonic immunoassay reader, the biosensor does not require any sophisticated experimental equipment, which makes it more accessible in the regions with limited resources.

## Additional File

Additional file 1: **Figure S1.** GOx concentration-dependent LSPR spectrum of AuNRs. LSPR spectrum of AuNRs etched by H_2_O_2_ from different concentrations of GOx. **Figure S2.** LSPR shift of plasmonic immunoassay for detection of different concentrations of Myo. LSPR shift of AuNRs based plasmonic immunoassay for detection of different concentrations of Myo. Each value presents the mean from 3 replicates. **Figure S3.** Calibration curve of AuNRs based plasmonic immunoassay for Myo detection. The calibration curve of plasmonic immunoassay for Myo detection is dependent on LSPR shift of AuNRs. Each value presents the mean from 3 replicates. **Figure S4.** Results of ELISA for detection of different concentrations of Myo. Each value presents the mean from 3 replicates. (DOCX 1858 kb)

## Abbreviations

Ab1: Anti-Myo antibody
Ab2: Anti-Myo antibody 2
AgNPRs: Triangular silver nanoprisms
ALS: Ambient light sensor
AMI: Acute myocardial infarction
AuNPs: Gold nanoparticles
AuNRs: Gold nanorods
ELISA: Enzyme-linked immunosorbent assay
GOx: Glucose oxidase
H_2_O_2_: Hydrogen peroxide
HRP: Horseradish peroxidase
LSPR: Localized surface plasmon resonance
Myo: Serum myoglobin
POCT: Point-of-care testing
