# Supplementary material for: Plasmonic ELISA for Sensitive Detection of Disease Biomarkers with a Smart Phone-Based Reader
Source: Nanoscale Res Lett. 2018 Dec 5;13:397. doi: 10.1186/s11671-018-2806-9 (PMC6281541; doi:10.1186/s11671-018-2806-9)
Supplement: Supplementary file 1 — Figure S1. GOx concentration-dependent LSPR spectrum of AuNRs. LSPR spectrum of AuNRs etched by H2O2 from different concentrations of GOx. Figure S2. LSPR shift of plasmonic immunoassay for detection of different concentrations of Myo. LSPR shift of AuNRs based plasmonic immunoassay for detection of different concentrations of Myo. Each value presents the mean from 3 replicates. Figure S3. Calibration curve of AuNRs based plasmonic immunoassay for Myo detection. The calibration curve of plasmonic immunoassay for Myo detection is dependent on LSPR shift of AuNRs. Each value presents the mean from 3 replicates. Figure S4. Results of ELISA for detection of different concentrations of Myo. Each value presents the mean from 3 replicates. (DOCX 1858 kb) [file 11671_2018_2806_MOESM1_ESM.docx]

**Preparation of anti-Myo antibody**

6-week-old female BALB/c mice were immunized with human Myo on days 0, 14, and 28. The method for screening monoclonal antibodies against Myo was established by using the cell fusion technology, indirect ELISA and competitive ELISA. Hybridomas synthesizing and secreting Myo-specific antibodies were subcloned twice by limiting dilution. Positive hybridoma clones were subcultured and frozen. The cell lines coded as 2G1 and 5G7 were selected to produce antibodies by injecting 10^6^ hybridoma cells in mice. The mAbs were precipitated with saturated ammonium sulfate and dissolved in phosphate buffer (0.002 M, pH 7.4) and then were dialyzed against the same buffer at 4°C for 24h. The IgG proteins were isolated by affinity chromatography with an immobilized protein G column. The antibody concentrations were determined by using the BCA protein assay.

**HRP-based ELISA for Myo detection**

For the HRP-based ELISA to detect Myo, we used the same antibody and classic double antibody sandwich ELISA to measure the levels of Myo in foetal bovine serum and clinical samples. Briefly, 96-well polystyrene plates were modified with Ab1 diluted in PBS at 4°C overnight. After washing the plates three times with PBST (1% Tween 20 in PBS), the plates were blocked with blocking buffer(1mg mL^-1^ BSA in PBS) at 37°C for 1h. Subsequently, the plates were washed three times with PBST, and Myo was added to the desired final concentration by diluting foetal bovine serum. After 1h incubation, the plates were washed three times with PBST, and 0.01 mg mL^-1^ Ab2–HRP was added at 37°C for 1h. After another washing step, 100μl TMB solution was added and incubated for 15 min at room temperature. At last, the reaction was terminated with 50 μl stop solution and the absorbance was measured at 450 nm.

**Preparation of Ab2-Sulfo-SMCC-GOx**

The Crosslinking of Ab2 and GOx with sulfo-SMCC was performed according to the Manufacturer’s instruction. Briefly, add 20 fold molar excess of Sulfo-SMCC solution to the Ab2 protein solution, allow reaction to proceed for 2 hours at 4℃.Then desalt activated protein sample to remove residual crosslinker through dialysis and add excessive GOx to the solution, reaction at 4℃ for 2 hours, then remove unconjugated proteins through ultrafiltration.

**Results**



**GOx concentration-dependent LSPR spectrum of AuNRs**

**Figure S1.** LSPR spectrum of AuNRs etched by H_2_O_2_ from different concentrations of GOx.


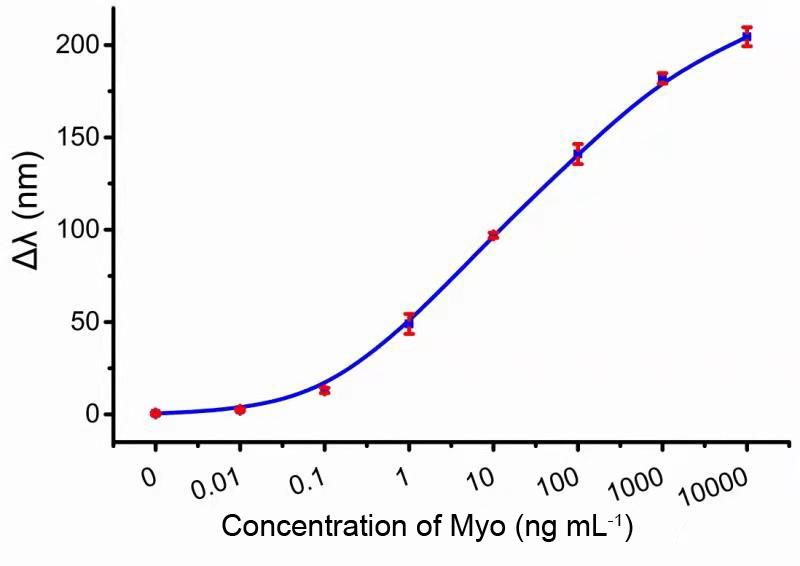
**LSPR shift of plasmonic immunoassay for different concentration of Myo**

**Figure S2.** LSPR shift of AuNRs based plasmonic immunoassay for detect different concentrations of Myo. Each value presents the mean from 3 replicates.


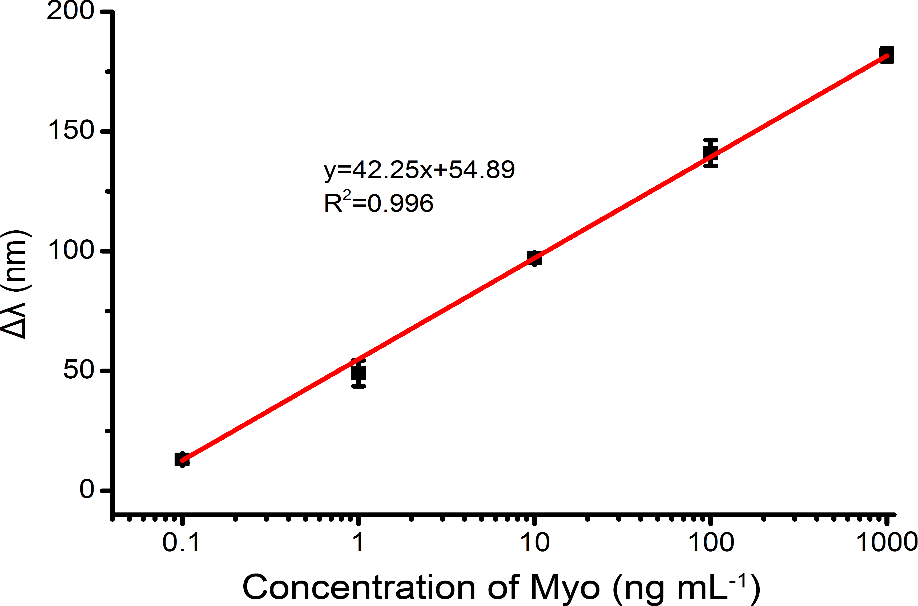
**Calibration curve of AuNRs based plasmonic immunoassay for Myo detection**

**Figure S3.** Calibration curve plasmonic immunoassay for Myo detection dependent on LSPR shift of AuNRs. Each value presents the mean from 3 replicates.

**

Results of ELISA for detect different concentration of Myo**

**Figure S4.** Results of ELISA for detect different concentrations of Myo. Each value presents the mean from 3 replicates.
